# Psychomotor Performance in Blended Versus In-Person Emergency Medical Technician Training: Retrospective Cohort Study

**DOI:** 10.2196/94950

**Published:** 2026-10-27

**Authors:** Cheng-Heng Liu, Ming-Ju Hsieh, Yu-Ting Chen, Chih-Wei Yang

**Affiliations:** 1 Department of Medical Education National Taiwan University Hospital Taipei City Taiwan; 2 Department of Emergency Medicine National Taiwan University Hospital Taipei City Taiwan; 3 Institute of Health Policy and Management National Taiwan University College of Public Health Taipei City Taiwan; 4 Department of Living Sciences National Open University New Taipei City Taiwan; 5 Graduate Institute of Medical Education and Bioethics National Taiwan University College of Medicine Taipei City Taiwan

**Keywords:** blended learning, emergency medical services, health professions education, psychomotor skills, retrospective cohort study, technology acceptance

## Abstract

**Background:**

Blended learning is common in health professions education, but aggregate comparisons may obscure differences among knowledge, procedural skill, and integrated performance. Whether these domains differ after program-level changes under a common assessment system remains uncertain.

**Objective:**

We aimed to compare knowledge, psychomotor performance, and integrated scenario competence between blended and traditional in-person emergency medical technician level 1 certification training, and, as an exploratory secondary aim, to describe digital engagement and technology acceptance among blended-cohort participants who completed the survey.

**Methods:**

We compared routinely collected certification records from traditional in-person (2020; n=603) and blended (2023; n=581) emergency medical technician level 1 cohorts. The primary outcome was psychomotor performance; secondary outcomes were knowledge, integrated scenario performance, and competency-threshold attainment. We estimated between-cohort differences with 95% CIs and adjusted linear regression. Equivalence was assessed using two 1-sided tests with expert-derived educational importance margins established through an instructor consensus exercise before the panel was provided with the between-cohort results (±5 points for knowledge, ±3 points for psychomotor performance, and ±4 points for integrated scenario performance). These margins represent educational consensus thresholds rather than externally validated clinical thresholds. Blended-cohort trainees were additionally offered a questionnaire on digital engagement and technology acceptance, analyzed by multiple linear regression on intention to use e-learning again.

**Results:**

Psychomotor performance was lower in the blended cohort (80.60, SD 9.70, vs 84.44, SD 10.80; mean difference 3.84, in-person minus blended, 95% CI 2.67-5.01; *d*=0.37; *P*<.001); the adjusted coefficient was −4.04 (95% CI −5.15 to −2.92; *P*<.001). Equivalence within the prespecified ±3-point margin was not demonstrated. Knowledge (difference −0.30, 95% CI −1.68 to 1.08) and integrated scenario performance (−0.94, 95% CI −1.89 to 0.00) fell within the prespecified ±5- and ±4-point margins, respectively. Psychomotor-threshold (521/581, 89.7%, vs 557/603, 92.4%; *P*=.10) and overall-certification rates (459/581, 79%, vs 474/603, 78.6%; *P*=.87) did not differ detectably. Knowledge-threshold attainment was higher in the blended cohort (516/581, 88.8%, vs 503/603, 83.4%; *P*=.007). Among the 236 blended-cohort participants who completed the survey, perceived benefits and positive attitudes toward digital learning were positively associated with intention to use e-learning again.

**Conclusions:**

The blended cohort had lower early psychomotor scores, whereas knowledge and integrated scenario differences fell within the prespecified margins. Programs adopting comparable models should assess psychomotor outcomes directly rather than infer them from knowledge or overall certification outcomes.

## Introduction

### Background

Blended learning is now a standing design choice in health professions education rather than a contingency, and the relevant question has shifted from whether programs can deliver content online to how different competencies perform after a program-level transition [1]. Pandemic-era transitions that prompted widespread adoption [2,3] retain methodological interest because they produced externally mandated changes applied to whole programs, a configuration difficult to obtain deliberately. Syntheses in health professions education report that blended learning yields knowledge outcomes at least comparable, and in some settings superior, to traditional instruction [1,4]. That evidence base, however, is dominated by aggregate comparisons of overall effectiveness. Whether a bundled program-level transition is associated differently with knowledge, procedural skill, and integrated performance under a common assessment system remains uncertain.

Health professions education is inherently multidimensional, requiring the integration of knowledge, psychomotor skills, and complex situational judgment. Traditional in-person health professions education has historically emphasized extensive hands-on practice, immediate instructor feedback, and scenario-based learning to support these competencies. Blended learning redistributes activities across digital and in-person environments [5,6]. In the program studied here, theoretical instruction moved online and scheduled practical hours were consolidated into fewer onsite course days. Evidence on psychomotor performance under such designs is inconsistent and often limited by small sample sizes, heterogeneous assessments, or reliance on self-reported competence [7]. Large-scale comparative data on standardized procedural and complex integrated performance outcomes remain sparse. A comprehensive evidence map of health professions digital education identified modality comparison, skills performance assessment, and learner-context interactions as areas with substantial remaining research gaps [8].

Another gap in the health professions education literature concerns learner digital readiness and technology acceptance, which may shape engagement with blended learning environments. The technology acceptance model and related frameworks hold that perceived usefulness and ease of use shape attitudes and intentions toward digital learning tools [9,10]. A systematic review of these applications in medical education found growing use alongside a need for context-specific validation across learner populations [11]. Digital literacy and self-efficacy are associated with engagement and performance in online learning [12,13], and inequalities in digital readiness have been documented in online education settings [14]. Emergency medical technician (EMT) trainees vary widely in age, educational background, and technological familiarity, and how these characteristics relate to engagement with blended learning has rarely been described in this population. The ancillary survey reported here addresses that descriptive gap among blended-cohort participants who completed the survey. It does not measure the certification outcomes and cannot account for any difference between the cohorts.

### Theoretical Framework

Blended learning can be understood as a form of instructional restructuring that alters when and how learning activities occur across competency domains. Three bodies of literature are summarized below, and they do not serve the same purpose. Cognitive load theory [15] and mastery learning and deliberate practice [16,17] provide a rationale for reporting the competency domains separately. Technology acceptance [9,10] does not bear on the comparison between domains and is used only for the exploratory secondary aim concerning digital engagement among blended-cohort participants who completed the survey.

Cognitive load theory describes why early procedural learning is demanding: declarative knowledge and motor execution must be coordinated while both are still unfamiliar [15]. It provides a rationale for reporting the 3 competency domains separately rather than as a composite, but it does not specify the direction of any particular change in sequencing.

Distributed access to theoretical content may alter opportunities for self-paced review and retrieval practice, which can strengthen retention [18]. Whether these opportunities translate into measurable differences in simulated clinical scenarios has not been established.

Mastery learning and deliberate practice frameworks bear on how instructional restructuring may influence procedural competence [16,17]. These models hold that expertise develops through repeated, feedback-rich practice until predefined standards are met. Where a program alters the timing or density of practice, initial performance and later retention may be affected, although the direction is not obvious a priori. A systematic review reported advantages for distributed rather than massed surgical-skills training in several outcomes [19]. A small quasi-experimental study compared 3-month retention after combined spacing and testing with retention after single-session training without testing [20]; because both spacing and testing differed, it does not isolate the contribution of either component. In the present setting, theoretical instruction was separated from practical sessions and the scheduled practical hours were distributed across fewer course days; these bundled changes cannot be distinguished by this study. Whether the consolidated onsite schedule changed how procedural practice was distributed cannot be determined from the available records.

Together, these perspectives justify domain-specific reporting but do not predict a specific ordering across assessment scales. The present study does not test the instructional mechanisms they propose.

Learner engagement with blended environments is also shaped by technology acceptance. Perceived usefulness and perceived ease of use are constructs of the technology acceptance model [10,21], while the facilitating-conditions construct comes from the unified theory of acceptance and use of technology [9]; digital literacy, self-efficacy, and experience further shape engagement behavior and perceived instructional value [12,13,22,23]. These constructs supplied the questionnaire items and organized the descriptive results reported for the exploratory secondary aim. They do not explain the certification outcomes because no acceptance measure was used as a predictor of assessment performance and the questionnaire was not administered to the traditional cohort.

The COVID-19–related transition from fully in-person to blended emergency medical technician level 1 (EMT-1) training at our institution created an opportunity for a retrospective historical cohort comparison of certification outcomes under a single national certification system. We compared knowledge, psychomotor performance, and integrated scenario competence between the traditional and blended cohorts, asking whether the difference between cohorts was of similar size across the 3 domains. As an exploratory secondary aim, we described digital engagement and technology acceptance among blended-cohort participants who completed the survey and examined which participant-reported factors were associated with their intention to use e-learning again; this second aim is confined to those participants and is not used to explain the between-cohort differences addressed by the first. The comparison is at the level of outcomes. It does not measure the learning processes that might explain any difference observed.

## Methods

### Study Design

We conducted a retrospective historical cohort comparison following an institutional change in instructional delivery related to the COVID-19 pandemic. The transition restructured EMT-1 training from a fully in-person format to a blended model. The curriculum framework, competency standards, and assessment procedures in both periods were governed by the same national certification system. The delivery format was fixed by program period and applied to every trainee enrolled in that period; there was no within-period choice between formats. This does not eliminate selection into course timing, attendance-related selection, or secular differences between the historically separated cohorts. This study analyzed routinely collected administrative training records from a regional EMT-1 training center; no prospective participant enrollment was conducted.

Two cohort periods were included: a pretransition cohort trained between January and December 2020 (traditional format) and a posttransition cohort trained between January and December 2023 (blended format). Cohorts trained in 2021 and 2022 were excluded because public health restrictions varied within and between course offerings, enrollment was reduced, and delivery format was not consistently defined. The 2023 cohort represents the earliest stable implementation of the blended curriculum, so the estimate may differ from one obtained after instructors had accumulated more experience with the format. This historical cohort design enabled comparison under one certification framework while retaining the limitations of a nonrandomized, historically separated cohort comparison.

### Setting and Educational Context

This study was conducted at a regional emergency medical services (EMS) education center accredited by Taiwan’s Ministry of Health and Welfare to deliver EMT-1 certification training. Taiwan’s national EMS training system follows a 3-tier structure (EMT-1, emergency medical technician level 2 [EMT-2], and EMT-Paramedic), with EMT-1 representing foundational training in basic life support, scene safety, patient assessment, and initial emergency interventions. The EMT-1 curriculum comprises 40 hours of standardized instruction, including didactic teaching, hands-on psychomotor skills training, and scenario-based simulation. Accredited training centers are required to implement a uniform national curriculum, adhere to predefined competency requirements, and apply standardized assessment tools. These requirements provide a common framework but do not by themselves establish comparable realized instruction across cohorts.

### Participants

This study’s population comprised trainee records available in the retained EMT-1 certification database for the designated study periods. Study eligibility criteria were age ≥20 years, completion of the required EMT-1 training components, attendance at ≥90% of scheduled instructional sessions, no prior EMT certification at any level, and recorded completion of the certification assessments. The retained administrative dataset contained 1251 trainee records after program-completion and attendance requirements had already been applied. Original course-enrollment rosters were unavailable, so the numbers initially enrolled and excluded before assembly of the retained dataset could not be determined for either cohort. Sixty-six records were subsequently excluded for age <20 years, leaving 1185 age-eligible records. Analyses then used a complete-case set: a trainee was included only if a score was recorded for all 3 certification assessments. One traditional-cohort record lacked a recorded psychomotor score and was excluded, leaving 1184 for analysis, comprising 603 in the traditional cohort and 581 in the blended cohort. The attendance criterion identifies trainees who completed within their own cohort. Under program regulations in force in both periods, trainees who missed scheduled sessions with approved leave could be deferred to a subsequent cohort, where they completed missed instruction and assessments within a 6-month window. In the blended program, attendance at live online sessions was verified by requiring cameras to remain on with the participant’s face identifiable; a designated assistant recorded attendance and captured periodic screenshots, and the live sessions were recorded. Trainees who missed live online hours were directed to the program’s make-up procedure before the onsite practical days. The available administrative records do not show how often this procedure was used and do not separate exclusions attributable to attendance, incomplete assessment, or prior certification, so cohort-specific exclusion counts and selection effects cannot be assessed.

### Instructional Conditions

#### Traditional In-Person Program (Pretransition Cohort)

The traditional cohort completed a 40-hour course delivered across 5 days and entirely in person. The schedule comprised 18 hours of didactic instruction and 22 hours of practical activities, including psychomotor skills practice and integrated scenarios. The supplied period-specific records did not independently document a 16-hour/6-hour subdivision of the practical component.

All sessions were conducted onsite. Instructor qualifications, competency standards, and assessment processes were governed by national EMT-1 requirements.

#### Blended Learning Program (Posttransition Cohort)

The blended cohort completed the same 40 scheduled instructional hours, reorganized so that the didactic component was delivered online and the practical component onsite. Eighteen hours of theoretical instruction were delivered online, comprising 4 hours of prerecorded material released before the course and 14 hours of live synchronous teaching via a videoconferencing platform with interactive elements. Session recordings were available for asynchronous review. The remaining 22 scheduled hours comprised face-to-face practical activities across 3 onsite course days, including psychomotor skills practice and scenario-based simulation; the supplied period-specific records did not independently document a 16-hour/6-hour subdivision. The recognized schedule in both periods totaled 45 hours, of which 5 were allocated to written and practical assessment; the 40 instructional hours reported throughout this paper exclude assessment time. The online component preceded the onsite days, but published course notices for 2023 show intervals ranging from 1 day to approximately 3 weeks across offerings. The records do not identify which offering each trainee attended.

### Documented Features of the Practical Training Component

The practical-training features are summarized in Table 1. The accredited provider had previously reported that, as a standing practice in both formats, learners practiced in pairs, classes were divided between concurrent skills stations, and instructors were assigned under a ceiling rule of 1 instructor per 15 learners. Because of rounding, the nominal ratio could range from approximately 1:10 to 1:15. The authors additionally confirmed from direct program knowledge that the onsite practical-training model, teaching materials, and instructor pool did not change between periods. These are program-level confirmations rather than period-specific session records. The records reviewed did not document realized group sizes, equipment used, cohort-specific scenario allocation, repetition counts, feedback or debriefing procedures, assessment-station configuration, or session-level instructor calibration.

Taken together, the provider’s standing-practice account and the authors’ direct program knowledge indicate that the onsite practical-training model, teaching materials, and instructor pool did not change between periods. The records reviewed did not permit a session-level audit of realized group size, equipment use, scenario allocation, repetition counts, feedback, debriefing, or hands-on exposure. The program transition nevertheless bundled online theory with consolidation of practical sessions into 3 onsite days rather than 5 course days. Observed differences therefore cannot be attributed to a single component.

The knowledge assessment (TR1), psychomotor skills assessment (TR2), and integrated scenario assessment (TR3) were administered at the conclusion of the training program, after all instructional components had been completed.

**Table 1:** Documented course and staffing information for the traditional and blended programs.

Parameter	Traditional in-person program (2020)	Blended program (2023)
Recognized schedule	45 hours (40 instructional, 5 assessment)	45 hours (40 instructional, 5 assessment)
Didactic instruction	18 hours, in person	18 hours, online (4 prerecorded, 14 live synchronous)
Scheduled practical instruction	22 hours	22 hours
Skills/scenario allocation	Not documented in supplied period-specific records	Not documented in supplied period-specific records
Delivery pattern	Five course days, with didactic and practical content partially interleaved within the same day	Online component preceding 3 onsite practical days; interval between components varied across offerings
Practice grouping	Learners in pairs; class divided between concurrent stations	Learners in pairs; class divided between concurrent stations
Instructor staffing	Same instructor pool; ceiling rule of 1 instructor per 15 learners	Same instructor pool; ceiling rule of 1 instructor per 15 learners
Facility, manikins, equipment	Same study center; period-specific room, manikin, and equipment records were not available for this audit	Same study center; period-specific room, manikin, and equipment records were not available for this audit
Scenario cases, repetition, and feedback	Teaching materials unchanged; cohort-specific scenario allocation, repetition counts, and feedback/debriefing records were not available for this audit	Teaching materials unchanged; cohort-specific scenario allocation, repetition counts, and feedback/debriefing records were not available for this audit
Assessment	Knowledge assessment (TR1^a^), psychomotor skills assessment (TR2^b^), and integrated scenario assessment (TR3^c^), under national protocols	Knowledge assessment (TR1), psychomotor skills assessment (TR2), and integrated scenario assessment (TR3), under national protocols

^a^TR1: knowledge assessment.

^b^TR2: psychomotor assessment.

^c^TR3: integrated scenario assessment.

### Outcome Measures

#### Primary Outcome

The primary outcome was psychomotor performance, assessed via TR2: a standardized skills assessment station evaluating out-of-hospital cardiac arrest management, including cardiopulmonary resuscitation quality metrics, airway management, and defibrillation procedures. The passing threshold was ≥70%.

#### Secondary Outcomes

Exploratory between-cohort comparisons were conducted for 2 additional certification assessments:

- TR1 (Knowledge): a multiple-choice examination scored out of 100 points, covering anatomy, physiology, pharmacology, and clinical decision-making (passing threshold: ≥60%).
- TR3 (Integrated Scenario Performance): a high-fidelity simulation requiring integration of cognitive and psychomotor competencies in randomly assigned scenarios selected from a standardized case bank (eg, acute myocardial infarction, pulmonary embolism, intracerebral hemorrhage, and prehospital out-of-hospital cardiac arrest triage and transport decisions; passing threshold: ≥70%).

All assessments were administered under the same national certification framework and the standardized operational procedures in effect during each study period. Historical interrater agreement estimates for the operational scoring rubrics were available from a separate association instructor calibration exercise conducted on September 28, 2020. During that exercise, evaluators independently scored the same recorded trainee performances, yielding κ=0.85 for TR2 and κ=0.82 for TR3. These estimates were not derived from duplicate ratings of the assessments analyzed in this study, which were routinely scored by a single evaluator. Because the original calibration forms and complete information on the numbers of raters and performances were unavailable, the estimates could not be recomputed, and their applicability to the assessments administered during the study periods could not be fully verified.

#### Ancillary Survey Instrument and Analysis

Digital learning experiences were assessed using a theory-informed, self-administered questionnaire offered to the blended cohort. The instrument measured digital engagement across 4 dimensions (skills, emotional, participation, and performance engagement) using 12 items adapted from Fredricks et al [24]. Perceived challenges (8 items), perceived benefits (5 items), and technology acceptance (4 items, based on technology acceptance model constructs) were also assessed, together with demographic characteristics and prior digital learning experience. Items adapted from English-language instruments were translated into Chinese and reviewed by the author team. The instrument showed high internal consistency across subscales (Cronbach α=.928-.941) and content validity established by expert review (content validity index=0.92). These survey outcomes are exploratory and are reported as such throughout. The questionnaire analysis included the 236 blended-cohort participants who completed the survey.

### Sample Size Calculation

Under equal group sizes, a 2-sided independent-samples *t* test with an α of .05 requires 176 participants per group to provide 80% power to detect a standardized mean difference (*d*) of 0.30. Both analyzed cohorts exceeded this requirement. Observed power is not reported because it is a deterministic function of the *P* values already given and adds nothing to the CIs. Nonsignificant results were not interpreted as evidence of equivalence [25]; equivalence testing against the prespecified margins is described in the Statistical Analysis subsection.

### Statistical Analysis

Statistical analyses were performed using SPSS (version 28.0; IBM Corp). Shapiro-Wilk tests rejected exact normality in all 6 cohort-by-outcome distributions (all *P*<.001). Given the large group sizes, between-cohort mean comparisons, education-stratified TR2 comparisons, and equivalence tests used pooled-variance (equal-variances-assumed) independent-samples t procedures and were interpreted using effect estimates and CIs. Effect sizes are reported as absolute Cohen d values; mean differences are reported with 95% CIs. Knowledge variances differed between cohorts (SD 13.30 vs 10.75); Welch *t* test for that comparison gave the same conclusion (difference −0.30, 95% CI −1.67 to 1.08; *P*=.67). The primary analyses used the same complete-case sample for all 3 outcomes (n=603 in the traditional cohort and n=581 in the blended cohort). An additional sensitivity analysis of TR3 included all available scenario scores (n=604 and n=581, respectively).

A nonsignificant difference is not evidence of equivalence [25]. Each outcome was therefore also assessed by two 1-sided tests against its prespecified educational importance margin, with equivalence declared only when both one-sided tests reached *P*<.05. Educational importance thresholds of 5 points for TR1, 3 points for TR2, and 4 points for TR3 were established through an in-person consensus discussion among 12 EMS instructors at the association’s instructor consensus camp on September 12, 2025. These thresholds were applied as symmetric equivalence margins of ±5, ±3, and ±4 points, respectively. The margins were established before the between-cohort analyses were conducted and before any study results were disclosed to the panel. Participants were asked to identify score differences that they considered educationally important for the knowledge, psychomotor, and integrated scenario assessments, and the discussion continued until consensus was reached. Based on the approximate scoring structure of the assessments, the margins were broadly comparable to the scores assigned to 2 knowledge-test items for TR1, 1 psychomotor checklist item for TR2, and either 1 major item or 2 minor items for TR3. These margins represent expert-derived educational thresholds rather than externally validated clinical thresholds (Multimedia Appendix 1). Competency-threshold attainment, defined as the proportion of trainees meeting the passing standard for each assessment and the proportion certified overall, was compared using Pearson chi-square tests without continuity correction. To assess whether the between-cohort imbalance in education level accounted for the psychomotor difference, that outcome was additionally examined within each education stratum. Sex differences were examined with 2-way ANOVA, fitted separately for each outcome with instructional modality, sex, and their interaction as factors and Type III sums of squares. The ANOVA interaction term tested whether sex differences varied by modality. Pearson correlation coefficients were calculated for age with each outcome within each cohort and for education level with each outcome, with education coded ordinally as high school or below, college or university, and graduate or higher. To assess the robustness of between-group differences, multivariable linear regression models were fitted for the primary outcome (TR2) and secondary outcomes (TR1, TR3) with instructional modality as the independent variable, adjusting for age, sex, and education level, using heteroscedasticity-consistent SEs (HC3 estimator). Education entered these models as a single ordinal term with the same 3 ordered categories. Regression coefficients (*b*) are unstandardized and are expressed in assessment score points. The survey data were analyzed separately from the certification outcomes, with the participant as the unit of analysis. Among the 236 blended-cohort participants who completed the survey, intention to use e-learning for future training was regressed on perceived benefits, positive attitudes toward digital learning, emotional engagement, performance engagement, and age in a single ordinary least squares model, with multicollinearity assessed using variance inflation factors. This model is exploratory and does not describe the full blended cohort.

Statistical significance was set at *P*<.05 for the primary outcome (TR2). TR1 and TR3 were treated as secondary exploratory comparisons; *P* values are reported descriptively without multiplicity correction.

### Ethical Considerations

This study’s protocol was reviewed and approved by the Research Ethics Committee B, National Taiwan University Hospital (NTUH-REC No. 202601212RINB), in accordance with the Declaration of Helsinki. Because this study involved retrospective analysis of deidentified educational records, the committee approved a waiver of informed consent. Data were deidentified at the source institution and stored in encrypted, password-protected databases in compliance with applicable data protection regulations.

## Results

### Participant Characteristics

The complete-case analysis included 1184 participants: 603 from the traditional cohort (January-December 2020) and 581 from the blended cohort (January-December 2023). All 3 assessments were administered as mandatory components of the certification program. Table 2 presents the baseline participant characteristics.

No statistically significant differences were detected in sex distribution (*P*=.19) or age (*P*=.54). Education level differed (*P*=.005), with a higher proportion of graduate-level trainees in the blended cohort (97/581, 16.7%, vs 68/603, 11.3%).

**Table 2:** Baseline participant characteristics.

Characteristic		In-person (n=603)	Blended (n=581)	*P* value	
Male, n (%)		325 (53.9)	291 (50.1)	.19	
Age (years), mean (SD)		31.2 (8.7)	31.5 (9.0)	.54	
Education level, n (%)					.005
	High school or below	141 (23.4)	104 (17.9)		
	College or university	394 (65.3)	380 (65.4)		
	Graduate or higher	68 (11.3)	97 (16.7)		

### Primary Outcome: Psychomotor Performance

Table 3 summarizes performance on the 3 assessments together with the equivalence tests against the prespecified margins.

Mean differences are calculated as in-person minus blended, and Cohen d values are absolute. Equivalence testing was conducted against the prespecified educational importance margins. “Met” indicates that both 1-sided tests reached a *P* value less than .05 under the stated numerical bound; it does not establish clinical equivalence. Because the 3 raw-score margins corresponded to different standardized widths (approximately 0.41, 0.29, and 0.48 SD for TR1, TR2, and TR3, respectively), the pattern of “met” and “not met” partly reflects margin width relative to score variability and should not be interpreted as a ranking of domain sensitivity.

Psychomotor performance was lower in the blended cohort (80.60, SD 9.70, vs 84.44, SD 10.80; mean difference 3.84, 95% CI 2.67-5.01; *P*<.001), with a small-to-medium effect size (*d*=0.37). The psychomotor outcome did not meet the prespecified equivalence criterion (upper 1-sided *P*=.92).

In a multivariable model adjusting for age, sex, and education level, the blended-cohort indicator was associated with lower psychomotor scores (*b*=−4.04, 95% CI −5.15 to −2.92; *P*<.001). The adjusted coefficient was slightly larger in magnitude than the unadjusted difference; the nonrandomized design does not permit a causal interpretation.

Because education level differed between cohorts, with a higher proportion of graduate-level trainees in the blended cohort, psychomotor performance was examined within each education stratum. The difference favoring the traditional cohort was present in all 3 strata. Values were 2.78 points (95% CI −0.32 to 5.89; *P*=.08) among trainees with high school education or below (n=141 vs 104), 4.15 points (95% CI 2.76-5.54; *P*<.001) among those with college or university education (n=394 vs 380), and 4.40 points (95% CI 1.55-7.25; *P*=.003) among those with graduate education (n=68 vs 97). The estimate was imprecise in the smallest stratum.

**Table 3:** Competency assessment outcomes by training modality.

Outcome	In-person, mean (SD)	Blended, mean (SD)	Difference (95% CI)	*|d|*	*P* value	Equivalence within margin
TR1^a^, knowledge	69.23 (13.30)	69.53 (10.75)	−0.30 (−1.68 to 1.08)	0.02	.67	Met (±5 points)
TR2^b^, psychomotor	84.44 (10.80)	80.60 (9.70)	3.84 (2.67 to 5.01)	0.37	<.001	Not met (±3 points)
TR3^c^, integrated scenario	85.59 (8.64)	86.53 (7.85)	−0.94 (−1.89 to 0.00)	0.11	.05	Met (±4 points)

^a^TR1: knowledge assessment.

^b^TR2: psychomotor assessment.

^c^TR3: integrated scenario assessment.

### Secondary Outcomes: Knowledge and Integrated Scenario Performance

Knowledge scores differed by −0.30 points between cohorts (95% CI −1.68 to 1.08; *d*=0.02; *P*=.67), and the difference fell within the prespecified ±5-point equivalence margin (both 1-sided *P*<.001). Integrated scenario scores differed by −0.94 points (95% CI −1.89 to 0.00; *d*=0.11; *P*=.05), and the difference fell within the prespecified ±4-point margin (both 1-sided *P*<.001). Using every record with a scenario score (n=604 vs 581), the difference was −1.08 points (95% CI −2.07 to −0.10; *d*=0.13; *P*=.03); the result still met the ±4-point margin. After covariate adjustment, neither knowledge (*b*=−0.21; *P*=.76) nor integrated scenario performance (*b*=0.62; *P*=.17) was associated with instructional modality. We draw no directional inference from the integrated scenario result.

### Competency-Threshold Attainment

Because certification depends on meeting fixed passing standards rather than on mean scores, threshold attainment was compared separately (Table 4). On the psychomotor assessment, 521 of 581 (89.7%) blended-cohort trainees and 557 of 603 (92.4%) traditional-cohort trainees met the 70% standard, a difference of −2.7 percentage points that did not reach significance (*P*=.10). Overall certification rates were 459 of 581 (79%) and 474 of 603 (78.6%), respectively (*P*=.87). Knowledge-threshold attainment was higher in the blended cohort (516/581, 88.8%, vs 503/603, 83.4%; *P*=.007), whereas integrated-scenario-threshold attainment did not differ detectably (559/581, 96.2%, vs 576/603, 95.5%; *P*=.55). The lower mean psychomotor score therefore did not correspond to a statistically significant between-cohort difference in meeting the national competency standard. The near-identical knowledge means, smaller SD, and 5.4-percentage-point higher pass rate in the blended cohort are consistent with a narrower score distribution in which fewer trainees fell below the 60-point threshold.

Overall certification is the certification outcome recorded in the training records.

**Table 4:** Competency-threshold attainment by training modality.

Assessment	Traditional in-person, n/N (%)	Blended, n/N (%)	Difference (blended minus in-person), percentage points^a^	*P* value
Knowledge (TR1^b^), ≥60	503/603 (83.4)	516/581 (88.8)	+5.4	.007
Psychomotor (TR2^c^), ≥70	557/603 (92.4)	521/581 (89.7)	−2.7	.10
Integrated scenario (TR3^d^), ≥70	576/603 (95.5)	559/581 (96.2)	+0.7	.55
Overall certification	474/603 (78.6)	459/581 (79.0)	+0.4	.87

^a^Positive values indicate a higher proportion in the blended cohort.

^b^TR1: knowledge assessment.

^c^TR2: psychomotor assessment.

^d^TR3: integrated scenario assessment.

### Demographic Associations With Performance

Age correlations were negative for all 3 outcomes in both cohorts. For knowledge, the correlations were r=−0.121 (*P*=.004) in the blended cohort and r=−0.055 (*P*=.17) in the traditional cohort. For psychomotor performance, they were r=−0.261 (*P*<.001) and r=−0.271 (*P*<.001), respectively; for integrated scenario performance, they were r=−0.158 (*P*<.001) and r=−0.150 (*P*<.001), respectively.

Female participants had higher mean scores in every cohort-by-outcome cell. In 2-way ANOVA models with sex and instructional modality as factors, the main effect of sex was significant for all 3 outcomes (TR1 *F*_1,1180_=48.0, TR2 *F*_1,1180_=15.7, and TR3 *F*_1,1180_=46.3; all *P*<.001). No sex-by-modality interaction was detected for TR1 (*F*_1,1180_=0.04; *P*=.84), TR2 (*F*_1,1180_=0.99; *P*=.32), or TR3 (*F*_1,1180_=0.18; *P*=.67).

Education level, coded ordinally as high school or below, college or university, and graduate or higher, was positively correlated with knowledge (r=0.147; *P*<.001) and with integrated scenario performance (r=0.130; *P*<.001). The correlation with psychomotor performance was near zero (r=0.021; *P*=.47). The education-stratified psychomotor difference persisted within every education stratum, including among trainees with graduate education, who were more numerous in the blended cohort. Knowledge-threshold attainment, which was higher in the blended cohort, was not examined within education strata.

### Secondary Outcomes: Digital Engagement and Technology Acceptance

Of the 236 blended-cohort participants who completed the survey (response rate: 236/581, 40.6%), approximately three-quarters (181/236, 76.7%) reported prior e-learning experience. No statistically significant differences were observed in digital engagement, perceived benefits, perceived challenges, or technology acceptance between participants with and without prior e-learning experience (all *P*>.05; Multimedia Appendix 2). In an exploratory multiple linear regression, perceived benefits (*b*=0.34; *P*<.001) and positive attitudes toward digital learning (*b*=0.28; *P*<.001) were positively associated with intention to use e-learning again. Emotional engagement and performance engagement were also associated with this intention, whereas age showed a modest negative association (*b*=−0.02; *P*=.047). The model explained 47% of the variance (R²=0.47; Multimedia Appendix 3). These findings describe survey participants only and are not generalized to the full blended cohort.

## Discussion

### Principal Findings

In this retrospective comparison of eligible completers from 2 certification cohorts assessed under 1 national system, a bundled program-level transition was associated with different outcome estimates across 3 competency domains. Psychomotor scores were 3.84 points lower in the blended cohort. Knowledge and integrated scenario differences were −0.30 and −0.94 points, respectively. The prespecified equivalence criterion was met for knowledge and integrated scenario performance and not met for psychomotor performance. These bounds were not validated clinical thresholds. Psychomotor-threshold, integrated-scenario-threshold, and overall-certification rates did not differ detectably; knowledge-threshold attainment was 5.4 percentage points higher in the blended cohort (*P*=.007). Among the 236 blended-cohort participants who completed the survey (236/581, 40.6%), perceived benefits and positive attitudes toward digital learning were positively associated with intention to use e-learning again. The survey findings are exploratory and describe survey participants only.

The score differences were 3.84 points for psychomotor performance, −0.30 for knowledge, and −0.94 for integrated scenario performance, each on its own assessment scale. Comparisons across domains are descriptive because the assessments are not on a common validated scale and no formal cross-domain contrast was performed. This study does not explain the observed estimates. Cognitive load, feedback quality, rehearsal continuity, and procedural integration were not measured, so the accounts discussed in the Comparison With Prior Evidence subsection are candidate explanations rather than findings.

These results also show why the components of a blended program should be described separately. In the equivalence tests, knowledge and integrated scenario scores met the prespecified margins, whereas psychomotor performance did not. Because delivery format, sequencing, calendar consolidation, and cohort year changed together, this study cannot identify which feature accounts for the observed pattern.

### Comparison With Prior Evidence

The knowledge-score difference was −0.30 points (95% CI −1.68 to 1.08), consistent with prior syntheses of online or blended vs face-to-face knowledge outcomes [1,4,26,27]. Reviews focused on clinical-skills outcomes address a related but distinct evidence base [7,28]. A broader online-learning meta-analysis also emphasized differences among instructional comparisons [29]. Knowledge and integrated scenario scores met the equivalence criteria within their prespecified margins.

Psychomotor performance differed between cohorts. Cognitive load theory offers one possible account [15], although this study did not measure cognitive load or schema formation. The distributed-practice literature addresses the more specific question of timing: a systematic review reported advantages for distributed rather than massed surgical-skills training in several outcomes [19], and a small quasi-experimental study compared 3-month retention after combined spacing and testing with retention after single-session training without testing [20]. Because spacing and testing changed together in the latter study, it does not identify the contribution of either component. Neither source tests the bundled separation of theory from practice and consolidation of practical hours in this program, and the present data do not identify a mechanism.

Two features of the blended program changed together and cannot be separated in this design: the interval between the online theory component and the first onsite practical session varied from 1 day to approximately 3 weeks across course offerings, and the practical hours were consolidated into 3 onsite days rather than distributed across 5 course days. Prior work identifies practice scheduling as a design consideration in procedural motor-skills training [30], but neither feature was manipulated independently here. An untested possibility is that a longer delay between the online theory component and the first onsite practical session would be associated with lower psychomotor scores. If so, trainees whose onsite days began the next day would be expected to score closer to the traditional cohort than trainees who waited approximately 3 weeks. These records do not permit that comparison, because no field links a trainee to a particular course offering, so the documented variation in interval cannot be joined to individual scores. Enrollment records identifying each trainee’s offering, or a prospective design in which the interval is assigned, would test the prediction. The observed difference therefore cannot be attributed to the theory-to-practice interval, the consolidated schedule, or any proposed learning process.

The wider literature on technology-supported resuscitation training is heterogeneous. Lin et al [31] reported little overall difference in chest-compression quality; among learners using feedback devices, only the appropriate compression-rate measure favored face-to-face training. Ko et al [32] found that the 3-practice blended arm had more correct-speed compressions and shorter hands-off time, and both blended arms performed better on environmental safety; knowledge, attitude, and confidence did not differ across groups. Boonmak et al [33] compared online with didactic theory after both groups received the same 2-hour skills training and reported comparable knowledge and skills outcomes. Iskander et al [34] established noninferiority for change in self-assessed confidence, not objective competence, among the 21% of participants with complete pre- and postcourse questionnaires. Sarboozi-Hosseinabadi et al [35] reported no statistically significant between-group differences at the 3 assessed time points and described webinar and simulation as equally effective. The remaining studies also differed in design and outcome: Bieri et al [36] assessed self-reported confidence and perceived knowledge without standardized psychomotor testing; Lim et al [37] found poorer overall skills with self-directed technology-based training without instructor guidance, while certainty across the reviewed evidence was very low; Chong et al [38] found no statistically significant adjusted differences in manikin scores, although the remote group practiced on more days and made more assessment attempts; and Chien et al [39] reported no significant difference between 6-month blended and traditional refresher groups, whereas skills declined more after a 12-month interval. Unlike their refresher single-skill context, our study examined initial multiskill certification training, so their comparison has limited direct applicability to learners acquiring several skills for the first time. These studies provide relevant comparisons, but they do not establish formal equivalence across delivery models or explain the between-cohort pattern observed here.

Integrated scenario scores fell within the prespecified ±4-point equivalence margin. Distributed access and flipped-classroom designs may support review, retrieval practice, and presession study [18,40], but this study did not test whether those processes affected scenario performance.

Age was modestly negatively correlated with psychomotor and integrated scenario performance in both cohorts. Although age has been examined in the technology-adoption literature [41], these performance correlations do not test a technology-adoption mechanism. The cohort-specific coefficients were numerically close (psychomotor: r=−0.261 in the blended cohort and r=−0.271 in the traditional cohort; integrated scenario: r=−0.158 and r=−0.150, respectively), but no formal age-by-cohort contrast was performed. These descriptive results therefore do not show whether delivery format modified the age association.

### Alternative Explanations

The 3-year interval between cohorts is the principal threat to internal validity. Familiarity with online instruction, expectations of remote work, and other unmeasured cohort characteristics may have differed between 2020 and 2023 and could have influenced outcomes independently of the program transition. Both cohorts completed the same 3 assessment domains under the same national certification protocols and passing standards. Period-specific item sets, checklists, and assessment scenarios were not available for this retrospective audit and therefore could not be verified as identical. This does not eliminate secular changes in learners or implementation, including how the same instructor pool delivered the course over time. The selective outcome pattern does not resolve this threat because temporal confounding could affect competency domains differently. We therefore cannot determine how much of the psychomotor difference reflects the bundled program transition or secular change.

A second explanation concerns delivery of the face-to-face component by the same instructor pool under a different calendar schedule. The blended program scheduled its 22 hours of practical activities across 3 onsite days, whereas the traditional program distributed the same scheduled practical hours across 5 course days. This study did not measure realized pacing, fatigue, cross-day revisiting, supervision, equipment use, feedback, or instructional quality. Because the transition bundled online delivery of theory with consolidation of practice, these components cannot be distinguished, and the observed difference is consistent with either or with other unmeasured implementation differences.

A third concern is attendance-based selection. The retained administrative dataset was assembled after program-completion and attendance requirements had already been applied. Because the original enrollment rosters and records of exclusions before dataset assembly were unavailable, we could not determine cohort-specific enrollment or exclusion counts. The direction and magnitude of any resulting selection bias therefore cannot be quantified.

### Implications for Instructional Design

The knowledge difference met the prespecified equivalence criterion, whereas the psychomotor difference did not. This contrast concerns observed outcomes and does not identify the responsible program component, because delivery format, practice scheduling, and cohort year changed together and the proposed instructional mechanisms were not measured. If the psychomotor difference reflects instructional sequencing, then programs adopting comparable formats may need to examine whether psychomotor training remains sufficiently intensive, feedback-rich, and temporally connected to theoretical learning; video-based skill previews, mental rehearsal, and greater density of early hands-on sessions are candidates for prospective testing.

### Theoretical Contribution

This study contributes to the literature on digital and blended health professions education by reporting 3 competency domains separately under 1 assessment system following a bundled program-level transition. Prior work has largely compared overall effectiveness; separate estimates avoid collapsing distinct score scales into a single summary judgment. The primary evidence therefore remains the estimated differences, their CIs, effect sizes, and competency-threshold results.

Separate reporting of competency domains may be useful in other procedural and simulation-based disciplines, including nursing, surgical training, and resuscitation education, as a prompt to disaggregate outcomes. More broadly, digital education interventions may warrant evaluation not only by aggregate outcomes but also by how different competencies perform.

### Limitations

This study has several limitations. The nonrandomized historical-cohort design prevents causal inference, and temporal confounding across the 3-year interval cannot be excluded. Period-specific assessment item sets, checklists, and scenarios were unavailable, so measurement comparability across years could not be verified. The exclusion of cohorts from 2021 and 2022, when delivery format varied under changing restrictions and enrollment was reduced, means that the comparison captures the earliest stable posttransition cohort and cannot describe instructor adaptation during the intervening years. The educational importance margins were based on a single instructor consensus discussion that included 3 study authors and were not independently validated thresholds. The reported κ values were historical interrater agreement estimates from a separate 2020 instructor calibration exercise rather than duplicate ratings of the analyzed assessments. Because the original calibration forms and complete information on the numbers of raters and performances were unavailable, the applicability of these estimates to the assessments administered during the study periods could not be verified.

Several implementation and selection features were also unmeasured. We did not collect measures of cognitive load, feedback quality, rehearsal continuity, or schema formation, so the proposed instructional mechanisms remain untested. The interval between online theory and the first onsite day varied from 1 day to approximately 3 weeks, but the records do not identify each trainee’s offering. Although the authors confirmed that the onsite practical-training model, teaching materials, and instructor pool did not change between periods, this program-level confirmation did not provide a session-level audit. Cohort-specific equipment use, scenario allocation, repetitions, feedback, debriefing, and assessment-station configuration were unavailable for retrospective verification. Cohort-specific exclusion counts and reasons were also unavailable, so attendance-based selection cannot be quantified.

Generalizability is limited by the single-center setting and by the ancillary survey, which was completed by 236 of 581 (40.6%) blended-cohort participants. The survey findings are exploratory, describe survey participants only, and cannot be generalized to the full blended cohort or used to explain the between-cohort differences. The outcome window was also narrow: we assessed immediate posttraining performance without retention or field-performance data, and technical skills decay at rates that differ by skill and initial proficiency [42]. Multicenter designs with longitudinal follow-up and direct measures of implementation and learning processes would address these gaps.

### Conclusions

In this single-center, nonrandomized comparison of cohorts 3 years apart, the institutional transition to blended delivery was associated with a 3.84-point lower early psychomotor score in the blended cohort (95% CI 2.67-5.01). The between-cohort differences were −0.30 points for knowledge (95% CI −1.68 to 1.08) and −0.94 points for integrated scenario performance (95% CI −1.89 to 0.00). The knowledge and integrated scenario differences met the prespecified equivalence criteria; the psychomotor difference did not. These educationally defined margins were not validated clinical thresholds.

Psychomotor-threshold, integrated-scenario-threshold, and overall-certification rates did not differ detectably between cohorts. Knowledge-threshold attainment was 5.4 percentage points higher in the blended cohort (*P*=.007).

Because delivery format, calendar consolidation, and cohort year changed together, this study cannot isolate the effect of moving didactic instruction online. The proposed instructional mechanisms were not measured and therefore remain candidate explanations rather than findings. Programs adopting comparable models should assess psychomotor outcomes directly rather than infer them from knowledge scores or overall pass rates. Whether denser early practice, shorter theory-to-practice intervals, or supplementary rehearsal would change outcomes requires prospective evaluation.

## Appendix

Multimedia Appendix 1 Source and development of the educational importance margins.

Multimedia Appendix 2 Survey subscale scores by prior e-learning experience (blended cohort, n=236).

Multimedia Appendix 3 Multiple linear regression model for future e-learning intentions (blended cohort, n=236).

## Abbreviations

EMS: emergency medical services
EMT: emergency medical technician
EMT-1: emergency medical technician level 1
EMT-2: emergency medical technician level 2
TR1: knowledge assessment
TR2: psychomotor skills assessment
TR3: integrated scenario assessment

## Data Availability

Deidentified data are available from the authors upon reasonable request.

## References

[1] Li M, Hong Y, Wu A, Ou W, Yang J, Lin G, Ruan Y, Chen Z, Lin C, Liang Y (2025). The effectiveness of blended learning in nursing and medical education: an umbrella review. Nurse Educ Pract.

[2] Rose S (2020). Medical student education in the time of COVID-19. JAMA.

[3] Thorne CJ, Kimani PK, Hampshire S, Hamilton-Bower I, Begum-Ali S, Benson-Clarke A, Couper K, Yeung J, Lockey A, Perkins GD, Soar J (2023). The nationwide impact of COVID-19 on life support courses. A retrospective evaluation by Resuscitation Council UK. Resusc Plus.

[4] Li C, He J, Yuan C, Chen B, Sun Z (2019). The effects of blended learning on knowledge, skills, and satisfaction in nursing students: a meta-analysis. Nurse Educ Today.

[5] Garrison DR, Kanuka H (2004). Blended learning: uncovering its transformative potential in higher education. Internet Higher Educ.

[6] Graham CR, Moore MG (2013). Emerging practice and research in blended learning. Handbook of Distance Education, 3rd ed.

[7] McGee RG, Wark S, Mwangi F, Drovandi A, Alele F, Malau-Aduli BS, ACHIEVE Collaboration (2024). Digital learning of clinical skills and its impact on medical students' academic performance: a systematic review. BMC Med Educ.

[8] Tudor Car Lorainne, Poon S, Kyaw BM, Cook DA, Ward V, Atun R, Majeed A, Johnston J, van der Kleij RMJJ, Molokhia M, V Wangenheim F, Lupton M, Chavannes N, Ajuebor O, Prober CG, Car J (2022). Digital education for health professionals: an evidence map, conceptual framework, and research agenda. J Med Internet Res.

[9] Venkatesh V, Morris MG, Davis GB, Davis FD (2003). User acceptance of information technology: toward a unified view. MIS Q.

[10] Davis FD (1989). Perceived usefulness, perceived ease of use, and user acceptance of information technology. MIS Q.

[11] Lee JWY, Tan JY, Bello F (2025). Technology acceptance model in medical education: systematic review. JMIR Med Educ.

[12] Prior DD, Mazanov J, Meacheam D, Heaslip G, Hanson J (2016). Attitude, digital literacy and self efficacy: flow-on effects for online learning behavior. Internet Higher Educ.

[13] Mohammadyari S, Singh H (2015). Understanding the effect of e-learning on individual performance: the role of digital literacy. Comput Educ.

[14] van de Werfhorst HG, Kessenich E, Geven S (2022). The digital divide in online education: inequality in digital readiness of students and schools. Comput Educ Open.

[15] Sweller J (1988). Cognitive load during problem solving: effects on learning. Cogn Sci.

[16] Ericsson KA, Krampe RT, Tesch-Römer C (1993). The role of deliberate practice in the acquisition of expert performance. Psychol Rev.

[17] McGaghie WC, Issenberg SB, Cohen ER, Barsuk JH, Wayne DB (2011). Does simulation-based medical education with deliberate practice yield better results than traditional clinical education? A meta-analytic comparative review of the evidence. Acad Med.

[18] Karpicke JD, Roediger HL (2008). The critical importance of retrieval for learning. Science.

[19] Jørgensen HK, Vamadevan A, Konge L, Hertz P, Bjerrum F (2025). Distributed training vs. massed practice for surgical skills training-a systematic review. Surg Endosc.

[20] Soares RV, Pedrosa RBDS, Sandars J, Cecilio-Fernandes D (2025). The importance of combined use of spacing and testing effects for complex skills training: a quasi-experimental study. Med Teach.

[21] Venkatesh V, Davis FD (2000). A theoretical extension of the technology acceptance model: four longitudinal field studies. Manage Sci.

[22] Eshet-Alkalai Y (2004). Digital literacy: a conceptual framework for survival skills in the digital era. J Educ Multimedia Hypermedia.

[23] Ng W (2012). Can we teach digital natives digital literacy?. Comput Educ.

[24] Fredricks JA, Blumenfeld PC, Paris AH (2004). School engagement: potential of the concept, state of the evidence. Rev Educ Res.

[25] Tomek J, Caldwell A, Eisner DA (2026). Beyond "non-significant" results: why and how to test for practical equivalence. Proc Natl Acad Sci U S A.

[26] Cook DA, Levinson AJ, Garside S, Dupras DM, Erwin PJ, Montori VM (2008). Internet-based learning in the health professions: a meta-analysis. JAMA.

[27] Ruiz JG, Mintzer MJ, Leipzig RM (2006). The impact of E-learning in medical education. Acad Med.

[28] McCutcheon K, Lohan M, Traynor M, Martin D (2015). A systematic review evaluating the impact of online or blended learning vs. face-to-face learning of clinical skills in undergraduate nurse education. J Adv Nurs.

[29] Means B, Toyama Y, Murphy R, Bakia M, Jones K (2009). Evaluation of evidence-based practices in online learning: a meta-analysis and review of online learning studies.

[30] Spruit EN, Band GPH, Hamming JF, Ridderinkhof KR (2014). Optimal training design for procedural motor skills: a review and application to laparoscopic surgery. Psychol Res.

[31] Lin L, Ni S, Cheng J, Zhang Z, Zeng R, Jin X, Zhao Y (2021). Effect of synchronous online vs. face-to-face cardiopulmonary resuscitation training on chest compression quality: a pilot randomized manikin study. Am J Emerg Med.

[32] Ko YC, Lin HY, Chiang WC, Yang CW, Hsieh MJ, Ma MHM (2024). Comparing the effects of blended learning and traditional instruction on basic life support for laypersons: a randomized controlled trial. J Formos Med Assoc.

[33] Boonmak S, Plailaharn N, Sripadungkul D, Somjit M, Gaysonsiri D, Boonmak P (2023). A randomized controlled, non-inferiority trial of Moodle online learning for basic life support training on learning outcomes among dental students. J Dent Educ.

[34] Iskander M, Haverkamp FJC, Geeraedts LMG, van Bilsen SJA, Tan ECTH (2026). Evaluation of a redesigned two-day trauma course incorporating blended learning compared with the traditional three-day trauma course: a retrospective non-inferiority study. World J Emerg Surg.

[35] Sarboozi-Hosseinabadi S, Sharifzadeh G, Hosseini SM (2024). Evaluating CPR training: simulation vs. webinars for Iranian emergency medical technicians during COVID-19. BMC Emerg Med.

[36] Bieri J, Tuor C, Nendaz M, Savoldelli GL, Blondon K, Schiffer E, Zamberg I (2023). Implementation of a student-teacher-based blended curriculum for the training of medical students for nasopharyngeal swab and intramuscular injection: mixed methods pre-post and satisfaction surveys. JMIR Med Educ.

[37] Lim XMA, Liao WA, Wang W, Seah B (2022). The effectiveness of technology-based cardiopulmonary resuscitation training on the skills and knowledge of adolescents: systematic review and meta-analysis. J Med Internet Res.

[38] Chong KM, Yang HW, He HC, Lien WC, Yang MF, Chi CY, Chen YP, Huang CH, Ko PC (2023). The effectiveness of online-only blended cardiopulmonary resuscitation training: static-group comparison study. J Med Internet Res.

[39] Chien CY, Tsai SL, Huang CH, Wang MF, Lin CC, Chen CB, Tsai LH, Tseng HJ, Huang YB, Ng CJ (2024). Effectiveness of blended versus traditional refresher training for cardiopulmonary resuscitation: prospective observational study. JMIR Med Educ.

[40] Chen F, Lui AM, Martinelli SM (2017). A systematic review of the effectiveness of flipped classrooms in medical education. Med Educ.

[41] Gagnon MP, Desmartis M, Labrecque M, Car J, Pagliari C, Pluye P, Frémont P, Gagnon J, Tremblay N, Légaré F (2012). Systematic review of factors influencing the adoption of information and communication technologies by healthcare professionals. J Med Syst.

[42] de Andres Crespo M, Lykoudis PM, Myint F, Berlingieri P (2025). Surgery and technical skill decay. Int J Surg.

