# Supplementary material for: Psychomotor Performance in Blended Versus In-Person Emergency Medical Technician Training: Retrospective Cohort Study
Source: JMIR Med Educ. 2026 Oct 27;12:e94950. doi: 10.2196/94950 (PMC13752586; doi:10.2196/94950)
Supplement: Supplementary file 1 [file mededu_v12i1e94950_app1.docx]

## **Source and Development of the Educational Importance Margins**

Educational importance thresholds of 5 points for TR1, 3 points for TR2, and 4 points for TR3 were established at an in-person instructor consensus camp held by the association on 12 September 2025. These thresholds were subsequently applied as symmetric equivalence margins of ±5, ±3, and ±4 points, respectively. Twelve EMS instructors participated in the discussion. Participants were asked to identify score differences that they considered educationally important for the knowledge, psychomotor, and integrated scenario assessments. The margins were established before the between-cohort analyses were conducted and before any study results were disclosed to the panel.

The discussion continued until the group reached consensus. No iterative rating rounds were conducted, and no formal voting threshold, individual ratings, or numerical agreement percentages were retained. Based on the approximate scoring structure of the assessments, the margins were broadly comparable to the scores assigned to two knowledge-test items for TR1, one psychomotor checklist item for TR2, and either one major or two minor items for TR3. Item-level assessment materials are not reproduced because they are restricted under the association’s regulations on assessment materials.

Table A presents the composition of the 12-member panel. The panel comprised 5 emergency physicians and 7 EMT-paramedics, with 5 to 24 years of EMS teaching experience. Names are presented as initials only, and years of teaching experience were provided by the association secretariat and reported as of September 2025. Three participants (CWY, CHL, and MJH) in the consensus exercise were coauthors of the present manuscript. The panel was therefore not independent of the study team. Although the margins were established before the panel was provided with the between-cohort results, they should be interpreted as author-involved educational consensus thresholds rather than externally validated clinical thresholds.

**Table S1.** Instructors who participated in the consensus discussion (n=12).

| **Initials** | **Professional role** | **Affiliation** | **Years of EMS teaching experience** |
| --- | --- | --- | --- |
| CWY | Emergency physician | National Taiwan University Hospital | 10 |
| WSY | EMT-P | Taipei City Fire Department | 24 |
| CHL | Emergency physician | National Taiwan University Hospital | 5 |
| WCC | EMT-P | Taipei City Fire Department | 17 |
| CYW | EMT-P | Kaohsiung Harbor Bureau | 18 |
| HHC | EMT-P | New Taipei City Fire Department | 10 |
| CTL | EMT-P | Taipei City Fire Department | 7 |
| CFH | EMT-P | Taipei City Fire Department | 19 |
| LWH | EMT-P | Taipei City Fire Department | 13 |
| YLC | Emergency physician | Taipei Tzu Chi Hospital | 8 |
| MJH | Emergency physician | National Taiwan University Hospital | 13 |
| JTS | Emergency physician | Far Eastern Memorial Hospital | 10 |
