# Supplementary material for: Psychomotor Performance in Blended Versus In-Person Emergency Medical Technician Training: Retrospective Cohort Study
Source: JMIR Med Educ. 2026 Oct 27;12:e94950. doi: 10.2196/94950 (PMC13752586; doi:10.2196/94950)
Supplement: Supplementary file 2 [file mededu_v12i1e94950_app2.docx]

## Survey Subscale Scores by Prior E-Learning Experience Among Blended-Cohort Participants

This appendix summarizes survey subscale scores for 236 blended-cohort participants (response rate: 236/581, 40.6%).

| Subscale | With Prior Experience (n=181), Mean (SD) | Without Prior Experience (n=55), Mean (SD) | ***P* value** |
| --- | --- | --- | --- |
| Digital Engagement | 3.82 (0.71) | 3.74 (0.68) | .43 |
| Perceived Benefits | 3.91 (0.82) | 3.78 (0.79) | .26 |
| Perceived Challenges | 2.45 (0.93) | 2.61 (0.88) | .22 |
| Technology Acceptance | 3.88 (0.76) | 3.72 (0.81) | .16 |

Note: Subscale scores are based on Likert-type items (1–5 scale). Digital engagement was measured across four dimensions (skills, emotional, participation, and performance engagement) using 12 items adapted from Fredricks et al. Perceived challenges included technical and non-technical barriers (8 items). Perceived benefits assessed educational value and efficiency gains (5 items). Technology acceptance measured attitudes and behavioral intentions based on TAM constructs (4 items). Independent-samples t-tests were used for between-group comparisons. No statistically significant differences were observed between learners with and without prior e-learning experience (all *P*>.05).

**References**

1. Fredricks JA, Blumenfeld PC, Paris AH. School engagement: potential of the concept, state of the evidence. Rev Educ Res. 2004;74(1):59-109. doi:10.3102/00346543074001059.

2. Davis FD. Perceived usefulness, perceived ease of use, and user acceptance of information technology. MIS Q. 1989;13(3):319-40. doi:10.2307/249008.

3. Venkatesh V, Morris MG, Davis GB, et al. User acceptance of information technology: toward a unified view. MIS Q. 2003;27(3):425-78. doi:10.2307/30036540.
