# Supplementary material for: Psychomotor Performance in Blended Versus In-Person Emergency Medical Technician Training: Retrospective Cohort Study
Source: JMIR Med Educ. 2026 Oct 27;12:e94950. doi: 10.2196/94950 (PMC13752586; doi:10.2196/94950)
Supplement: Supplementary file 3 [file mededu_v12i1e94950_app3.docx]

## Multiple Linear Regression Model for Future E-Learning Intentions Among Blended-Cohort Participants

The regression analysis included 236 blended-cohort participants (response rate: 236/581, 40.6%).

| Predictor | *b* | SE | t | ***P* value** | 95% CI |
| --- | --- | --- | --- | --- | --- |
| Constant | 0.82 | 0.31 | 2.65 | .009 | [0.21, 1.43] |
| Perceived Benefits (TB) | 0.34 | 0.07 | 4.86 | <.001 | [0.20, 0.48] |
| Positive Attitudes (EX) | 0.28 | 0.06 | 4.67 | <.001 | [0.16, 0.40] |
| Emotional Engagement | 0.19 | 0.08 | 2.38 | .02 | [0.03, 0.35] |
| Performance Engagement | 0.16 | 0.07 | 2.29 | .02 | [0.02, 0.30] |
| Age | −0.02 | 0.01 | −2.00 | .047 | [−0.04, 0.00] |

Model summary: R² = 0.47, Adjusted R² = 0.46, F(5, 230) = 40.82, *P*<.001

Note: Dependent variable = intention to use e-learning for future training. Multicollinearity was assessed using variance inflation factors (VIF < 3 for all predictors). b = unstandardized regression coefficient, expressed in units of the outcome scale; SE = standard error; CI = confidence interval. TB = total benefits subscale; EX = experience/attitudes subscale.
